# High-Dimensional DNA Methylation Mediates the Effect of Smoking on Crohn’s Disease

**DOI:** 10.3389/fgene.2022.831885

**Published:** 2022-04-05

**Authors:** Tingting Wang, Pingtian Xia, Ping Su

**Affiliations:** ^1^ Institute of Medical Sciences, The Second Hospital, Cheeloo College of Medicine, Shandong University, Jinan, China; ^2^ Department of General Surgery, Qilu Hospital, Cheeloo College of Medicine, Shandong University, Jinan, China

**Keywords:** epigenome wide, DNA methylation, mediation effect, causal diagram, smoking

## Abstract

Epigenome-wide mediation analysis aims to identify high-dimensional DNA methylation at cytosine–phosphate–guanine (CpG) sites that mediate the causal effect of linking smoking with Crohn’s disease (CD) outcome. Studies have shown that smoking has significant detrimental effects on the course of CD. So we assessed whether DNA methylation mediates the association between smoking and CD. Among 103 CD cases and 174 controls, we estimated whether the effects of smoking on CD are mediated through DNA methylation CpG sites, which we referred to as causal mediation effect. Based on the causal diagram, we first implemented sure independence screening (SIS) to reduce the pool of potential mediator CpGs from a very large to a moderate number; then, we implemented variable selection with de-sparsifying the LASSO regression. Finally, we carried out a comprehensive mediation analysis and conducted sensitivity analysis, which was adjusted for potential confounders of age, sex, and blood cell type proportions to estimate the mediation effects. Smoking was significantly associated with CD under odds ratio (*OR*) of 2.319 (95% CI: 1.603, 3.485, *p* < 0.001) after adjustment for confounders. Ninety-nine mediator CpGs were selected from SIS, and then, seven candidate CpGs were obtained by de-sparsifying the LASSO regression. Four of these CpGs showed statistical significance, and the average causal mediation effects (ACME) were attenuated from 0.066 to 0.126. Notably, three significant mediator CpGs had absolute sensitivity parameters of 0.40, indicating that these mediation effects were robust even when the assumptions were slightly violated. Genes (BCL3 and FKBP5) harboring these four CpGs were related to CD. These findings suggest that changes in methylation are involved in the mechanism by which smoking increases risk of CD.

## Introduction

Inflammatory bowel disease (IBD) is a complex etiology comprising Crohn’s disease (CD) and ulcerative colitis (UC) (Severs et al., 2016). Previous studies have shown that the relationship between smoking and IBD is complex and remains the most independent and prominent risk factor. It is well established that smoking has significant detrimental effects on the course of CD, but it has a beneficial influence on the development of UC (Tanja Birrenbach MUBM, 2004; Khasawneh et al., 2017; Nicolaides et al., 2021; van der Sloot et al., 2021). However, the efficacy of smoking on IBD remains largely unknown. Furthermore, it is less clear how smoking impacts the biological mechanism of CD.

DNA methylation has a role in the immune dysfunction phenotype associated with IBD, as it is influenced by certain smoking (Tsaprouni et al., 2014) known to be associated with inflammatory diseases (McDermott et al., 2015). DNA methylation is a crucial mechanism associated with environmental exposures, particularly smoking and alcohol (Lee and Pausova, 2013; Tsaprouni et al., 2014; Joehanes et al., 2016; Jenkins et al., 2017; Sharp et al., 2018; Zhang et al., 2018), and complex diseases such as rheumatoid arthritis, type 2 diabetes, and IBD (Liu et al., 2013; Ventham et al., 2016; Davegårdh et al., 2018). In epigenetic studies, it is of increasing scientific interest to study the mediating role of DNA methylation in the etiology of human diseases (Liu et al., 2013; Ventham et al., 2016; Zhang et al., 2016; Kular et al., 2018). Epigenome-wide association studies (EWASs) have explored associations of DNA methylation across the genome and identified epigenetic marks of disease (Dick et al., 2014; Wahl et al., 2017). Previous studies have focused on associations between DNA methylation and either exposure/outcomes, it is useful to test for mediation of the effect of exposure on outcome by DNA methylation (Fujii et al., 2021). Based on the causal inference, DNA methylation may act as potential mediator linking environmental exposure and disease outcomes. Recently, increasing evidence points towards a major role for epigenetic mechanisms of DNA methylation in regulating the fundamental behavior of CD. Studies have detected the links between 25 CpG sites and CD as well as the links between 13 CpG sites and UC with specific DNA methylation (Lin et al., 2011; Karatzas et al., 2014). However, limited data exist concerning the contribution of DNA methylation to CD pathogenesis. Epigenome-wide mediation analysis needs to be conducted in ultra-high-dimensional DNA methylation CpG sites simultaneously to explore statistically significant CpG sites. Karatzasa et al. showed the different known genes whose methylation has been related to IBD, CD, or UC, respectively (Karatzas et al., 2014). Recent work has been focused on researching associations between genetic risk and IBD through DNA methylation (Ventham et al., 2016). However, few studies have examined the role of smoking associated with DNA methylation on the development mechanism of CD.

DNA methylation is immensely cell-type specific, and several studies have demonstrated the impacts of cellular heterogeneity on the DNA methylation status (Liu et al., 2013; Jaffe and Irizarry, 2014; Inoshita et al., 2015; Shu et al., 2020), which may act as a potential confounder when investigating the effect of DNA methylation on disease. Therefore, we adjusted for confounders of age, sex, and blood cell type proportions to estimate the mediation effects. Currently, there is a focus on high-dimensional mediation analysis in epigenome-wide mediation. Based on the concept of SIS and regularization techniques (minimax concave penalty, MCP) in a high-dimensional mediation analysis, Zhang et al. (Zhang et al., 2016) established a HIMA model to identify DNA methylations mediating the relationship between smoking and lung function. In summary, CpG sites with DNA methylation that mediate the effect of smoking on CD to improve techniques for early disease detection and prevention are identified.

In this study, we identify the mediating effect of the association between smoking and CD through methylation mechanisms at CpG sites. We applied the multiple-mediator causal model framework to estimate and test unbiased mediation effects in high-dimensional epigenetic studies, in particular the existence of omitted variables or confounders. In the primary analysis, we first reduced the pool of potential mediator CpGs using the SIS (Fan and Lv, 2008) method and further conducted variable selection with the de-sparsified LASSO (Dezeure et al., 2014; van de Geer et al., 2014). By de-sparsifying the LASSO coefficients, one can reduce the estimation bias and obtain the asymptotic normality of the regression estimates. Finally, we implemented mediation analysis to assess the mediation effect of smoking on CD. We further estimated causal mediation effects and conducted sensitivity analysis for the possible existence of confounding by unmeasured covariates. Our results provide new insights into the role of DNA methylation in how smoking affects CD.

## Methods

### Subjects

Datasets were obtained from a case–control study of DNA methylation and IBD. The genome-wide DNA methylation data using the Illumina 450K methylation array are available at the Gene Expression Omnibus (GEO) website under accession GSE87648 to identify IBD-associated epigenetic analysis (Ventham et al., 2016). In our study, exposure was a binary variable, smoking (current versus former smokers or never smokers). After subjects with missing smoking status were excluded, the final dataset comprised 103 CD cases and 174 controls (symptomatic and healthy controls) with DNA methylation data available being used for mediation analyses (Ventham et al., 2016).

### Methylation Data

First of all, we carried out a series of quality control: probes with detection *p*-value (default ≥0.01) and samples with a mean *p*-value of all probes greater than 0.05 were filtered out; a total of 28,931 probes containing SNPs (MAF ≥ 0.05) in their sequences were also removed from the final data; and all probes located in chromosomes X and Y were filtered out. Meanwhile, for normalization of methylation data, a quantile normalization algorithm (Fortin et al., 2014) was the normalization method of the Illumina Infinium HumanMethylation450 platform to remove unwanted variation by regressing out variability explained by the control probes present on the array (Amiri Roudbar et al., 2020). The above-described methylation markers were standardized to ensure that the coefficients are in the same scale. After data preprocessing, 242,594 methylation sites were available for the downstream analysis. All methylation array data preprocessing was conducted with the R package minfi (Aryee et al., 2014). A study indicated that the M-value was more statistically accepted than the beta value for the differential methylation analysis (Du et al., 2010). Thus, the DNA methylation level was calculated as the M-value for our statistical analysis.

### Statistical Analysis

**FIGURE 1:**
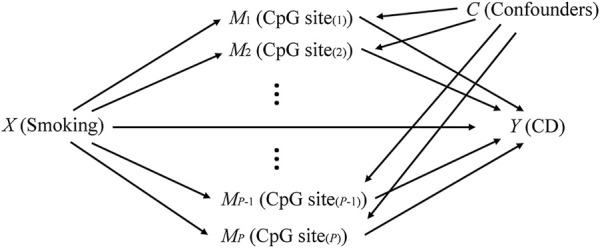
The causal diagram with high-dimensional mediators.

The causal graph model in Figure 1 assumed independence of multiple causal mechanisms in situations with confounders. *X* and *Y* represent exposure (smoking) and outcome (CD), respectively. ***M*** = (*M*_1_, …, *M*_*P*_) denotes high-dimensional mediators (CpG sites) that we are interested in for their effects independent of the pathway from exposure to outcome. Suppose that there are multiple causally unrelated mediators and that one is interested in estimating the causal mediation effects with respect to each of them. Let *C* denote some set of comprehensive confounders (sex, age, estimated CD8^+^ T cells, CD4^+^ T cells, natural killer (NK) cells, B cells, monocytes, and granulocytes) that may affect the mediator and outcome.

### Association Between Smoking and CD at Baseline

This analysis was conducted using baseline information on smoking, CD, and confounders (Figure 1). The following logistic regression model was used to test the association of smoking and CD, adjusting for confounders:

$$\begin{array}{l} \log\text{it}\left(P\right)=\beta_{0}+\beta_{1}\;smoking\;\;\;+\beta_{2}\;\;\;age+\beta_{3}\;sex+\beta_{4}\;CD8T+\beta_{5}\;CD4T+\\ \beta_{6}\;NKcell+\beta_{7}\;Bcell+\beta_{8}\;monocytes+\beta_{9}\;granulocytes \end{array}$$

### Mediator Screening and Mediation Effect Analysis

First, for the purpose of dimension reduction analysis, a high dimension may lead to false associations between covariates and response variables. We implemented SIS (Fan and Lv, 2008) to reduce the dimensionality of high-dimensional mediator CpG sites. Let $M_{*}=\left\{1\leq i\leq p:\beta_{i}\neq 0\right\}$ be the true sparse model with non-sparsity size $s=\text{ |}M_{*}|$ . And then let $\omega ={\left(\omega_{1},...,\omega_{p}\right)}^{\text{T}}$ , for any given γ∈(0,1) ; we sort the *p* componentwise magnitudes of the vector ω in decreasing order and define a submodel $M_{\gamma }=\left\{1\leq i\leq p:\left|\omega_{i}\right|\text{ is among the first }\left[\gamma n\right]\text{ largest of all}\right\}$ , where [γ*n*] denotes the integer part of γ*n* and [γ*n*] < *n*. The SIS method was used for a rough dimension reduction to reduce the ultra-high-dimensional model to *d* ( d≤  n ) dimension depending on the order of sample size *n* (*n* = 277). It was crucial that the SIS of a fast and efficient method reduce dimensionality from a large or huge scale to a relatively large scale. Therefore, to identify important mediators with the largest effects for the response, SIS identifies mediators of the top d=    2n/log(n) (Zhang et al., 2016) instead of d=    n/log(n) in Fan and Lv (2008).

Second, after dimensionality reduction of SIS, variable selection was carried out next. On account of the LASSO estimates being biased and without the testing to asymptotic normality property, previous studies proposed the asymptotic normality for the de-sparsified estimates for high-dimensional data (Dezeure et al., 2014; van de Geer et al., 2014). Dezeure et al. (2014) did a comprehensive method for high-dimensional inference to test the regression coefficient. The method was based on the asymptotic normality of de-sparsifying the LASSO regression to obtain the bias-corrected regression coefficient following van de Geer et al. (2014), and furthermore, we could get a *p*-value for each mediator. De-sparsifying the LASSO procedure has been implemented in R package hdi (Dezeure et al., 2014). In the paper, we described the de-sparsifying approach for a binary outcome *CD*. Let $\rho_{\beta }\left(y,x\right)=\rho \left(y,x\beta \right)$ was a loss function, and define $\;\;{\dot{\rho }}_{\beta }:=\frac{\partial }{\partial \beta }\rho_{\beta }$ and ${\ddot{\rho }}_{\beta }:=\frac{\partial^{2}}{\partial \beta \partial \beta^{T}}\rho_{\beta }$ , and further define $P_{n}g=\sum_{i=1}^{n}g\left(y_{i},\;x_{i}\right)/n$ . The LASSO estimator for the CpG coefficients *β* was given as $\hat{\beta }=\text{arg min}\left(P_{n\rho_{\beta }}+\lambda {\left\|\beta \right\|}_{1}\right)$ , where λ was a tuning parameter. Define $\hat{\Sigma }:=P_{n}{\ddot{\rho }}_{\hat{\beta }}$ and construct $\hat{\Theta }=\;{\hat{\Theta }}_{\text{LASSO}}$ by doing a nodewise LASSO with $\hat{\Sigma }$ as input. Then the de-sparsified LASSO estimator was given as $\hat{b}:=\hat{\beta }-\hat{\Theta }P_{n}{\ddot{\rho }}_{\hat{\beta }}$ . van de Geer et al. (2014) gave the detailed algorithm for computing the de-sparsified LASSO estimators in a generalized linear model framework. It was crucial that the method under the generalized linear model could reduce the estimation bias and obtain the asymptotic normality of the regression estimates (van de Geer et al., 2014). Furthermore, we could obtain a *p*-value for each CpG site based on the asymptotic normality of the de-sparsified LASSO estimates. Studies of van de Geer et al. (2014), Dezeure et al. (2014), and Wu et al. (2018) provided detailed information about the de-sparsified LASSO estimates. Meanwhile, we corrected the multiple testing by using a false discovery rate (*FDR*) of 5% (<0.05).

Finally, with the reduced dimension, the below-described procedures can be followed to assess the mediation effect. Among the selected mediators, we estimate the average direct effects (ADE) and the average causal mediation effects (ACME) of the mediator based on the mediation package in R (Tingley et al., 2014). To assess the robustness of the results if the sequential ignorability (SI) assumption was violated, we conducted a sensitivity analysis developed by Imai et al. (2010a). Our article assumes the following as regards SI: (1) it is conditional on the covariates, and the exposure is independent of all potential values of the outcome and mediator; and (2) the observed mediator is independent of all potential outcomes given the observed exposure and covariates (Imai et al., 2010b; Imai and Yamamoto, 2013; Shu et al., 2020). The sensitivity parameter is the correlation *ρ* between the residuals of the mediator and outcome regressions (Imai et al., 2010a). For each mediator, sensitivity plots were illustrated to show the estimated ACME and their 95% confidence interval as a function of *ρ*. If the *ρ* at which ACME = 0 was close to 0, it indicates that the mediation analysis was sensitive to violation of the SI assumption.

In mediation analysis, for each candidate CpG mediator, we fitted the following statistical models: (1) the mediator model, the CpG site (*M*) as the outcome and smoking (*X*) as a predictor, adjusting for the confounders sex, age, and estimated cell-type proportions; and (2) the outcome model, with CD (*Y*) as the outcome and smoking (*X*) as a predictor, adjusting for the mediator, i.e., the CpG site (*M*), and the covariates from the first model.

The Mediator Model Was Fit

$$\begin{array}{l} E\left[M|x,c\right]=\beta_{0}+\beta_{1}x+\beta_{2}\;\;\;age+\beta_{3}\;sex+\beta_{4}\;CD8T+\beta_{5}\;CD4T+\\ \;\;\;\beta_{6}\;\;NKcell+\;\beta_{7}\;\;Bcell+\beta_{8}\;\;monocytes+\beta_{9}\;\;granulocytes\;\;\;\;\;\;\;\;\;\;\;\\ \log it\left\{P\left(Y=1|x,m,c\right)\right\}=\theta_{0}+\theta_{1}x+\theta_{2}m+\theta_{3}\;\;age+\theta_{4}\;\;sex+\theta_{5}\;CD8T+\\ \theta_{6}\;CD4T+\theta_{7}\;NKcell+\;\theta_{8}\;Bcell+\theta_{9}\;monocytes+\theta_{10}\;granulocytes\;\;\;\;\;\;\;\; \end{array}$$

Then the ADE and ACME odds ratios are given by VanderWeele and Vansteelandt (2014):

$$\begin{array}{l} \log\left(OR^{ADE}\right)=\theta_{1}\\ \log\left(OR^{ACME}\right)=\beta_{1}\theta_{2} \end{array}$$

The estimates and 95% confidence intervals were estimated by nonparametric bootstrapping with 1,000,000 iterations (Tingley et al., 2014).

## Results

The distribution of demographic and clinical characteristics based on baseline case–control status is summarized in Table 1. A total of six different cell types including two types of T cells (CD8^+^ T cells and CD4^+^ T cells), NK cells, B cells, monocytes, and granulocytes. Table 1 shows that the cell-type proportions (CD8^+^ T cells, CD4^+^ T cells, NK cell, B cell, monocyte, and granulocyte) for each of the samples were estimated using the estimateCellCounts function implemented in a flexible and comprehensive bioconductor “Minfi” (Aryee et al., 2014), which obtained sample-specific estimates of cell proportions based on reference information on cell-specific methylation signatures (Houseman et al., 2012).

**TABLE 1:** Sample characteristics and differential cell-type proportions.

Variables	Controls (n = 174)	Cases (n = 103)	*z*	*p*-value
Age	35.667 (±12.459)	38.738 (±16.266)	−1.651	0.101
Smoking (N, %)			17.394	<0.001
Current	38 (0.218)	48 (0.466)		
Never/Ex	136 (0.782)	55 (0.534)		
Sex (N, %)			0.012	0.912
Male	87 (0.5)	53 (0.515)		
Female	87 (0.5)	50 (0.485)		
CD8^+^ T cells	0.104 (±0.046)	0.067 (±0.043)	6.521	<0.001
CD4^+^ T cells	0.147 (±0.062)	0.101 (±0.069)	5.675	<0.001
NK cells	0.04 (±0.038)	0.024 (±0.037)	3.346	0.001
B cells	0.081 (±0.033)	0.06 (±0.026)	5.684	<0.001
Monocytes	0.065 (±0.022)	0.067 (±0.028)	−0.693	0.489
Granulocytes	0.599 (±0.11)	0.71 (±0.124)	−7.755	<0.001

The mean age of cases was 38.738 (standard deviation (SD), 16.266) years, which is older than that of the controls by 3 years. There was no significant statistical difference between the age and sex. On average, controls had a higher proportion of CD8^+^ T cells, CD4^+^ T cells, NK cells, and B cells. Compared to controls, a larger proportion of cases were granulocytes cells. Notably, there was a significant difference in smoking.

### Association Between Smoking and CD at Baseline

We found that the effect of smoking on the CD using the logistic regression model remained significant with an odds ratio (*OR*) of 2.319 (95% CI: 1.603, 3.485, *p* < 0.001), adjusting for sex, age, CD8^+^ T cells, CD4^+^ T cells, NK cells, B cells, monocytes, and granulocytes (Table 2). The results suggested that smoking accelerated CD progression, which was consistent with previous reports (Nicolaides et al., 2021; van der Sloot et al., 2021).

**TABLE 2:** The estimation of smoking by logistic regression.

Variable	Estimation	*SE*	*OR* (95% CI)	*p*
Smoking	0.841	0.191	2.319 (1.603, 3.485)	<0.001

SE, standard error

### Dimensionality Reduction and Mediation Analysis

A total of    d=2n/log(n)=99 CpGs met our candidate selection through the SIS method. As shown in Table 3, the results of de-sparsifying the LASSO showed seven CpGs by multiple testing correction, i.e., an *FDR* of *P*_*FDR*_ < 0.05 in models adjusted for sex, age, CD8^+^ T cells, CD4^+^ T cells, NK cells, B cells, monocytes, and granulocytes. The effect size of CpGs was positive (cg04287259 and cg05941027) or negative (cg25114611, cg10180440, cg19821297, cg26470501, and cg09349128), but the absolute effect value was greater than 2. The standard error (SE, i.e., prediction accuracy) from de-sparsifying the LASSO method was relatively small, varying from 0.6791 to 1.253.

**TABLE 3:** The correction *p*-value of de-sparsifying the LASSO method.

CpG	*p*	*FDR* ^a^	Estimation	95% CI	*SE*
cg04287259	0.001	0.031	3.123	(1.202, 5.051)	0.982
cg25114611	0.001	0.031	−3.279	(−5.227, −1.332)	0.994
cg10180440	0.003	0.042	−2.040	(−3.372, −0.707)	0.680
cg05941027	0.003	0.042	2.478	(0.870, 4.086)	0.820
cg19821297	0.001	0.031	−2.169	(−3.499, −0.839)	0.679
cg26470501	<0.001	0.031	−4.486	(−6.941, −2.030)	1.253
cg09349128	0.001	0.031	−2.441	(−3.932, −0.951)	0.761

SE, standard error.

a FDR-adjusted *p*-value.

**FIGURE 2:**
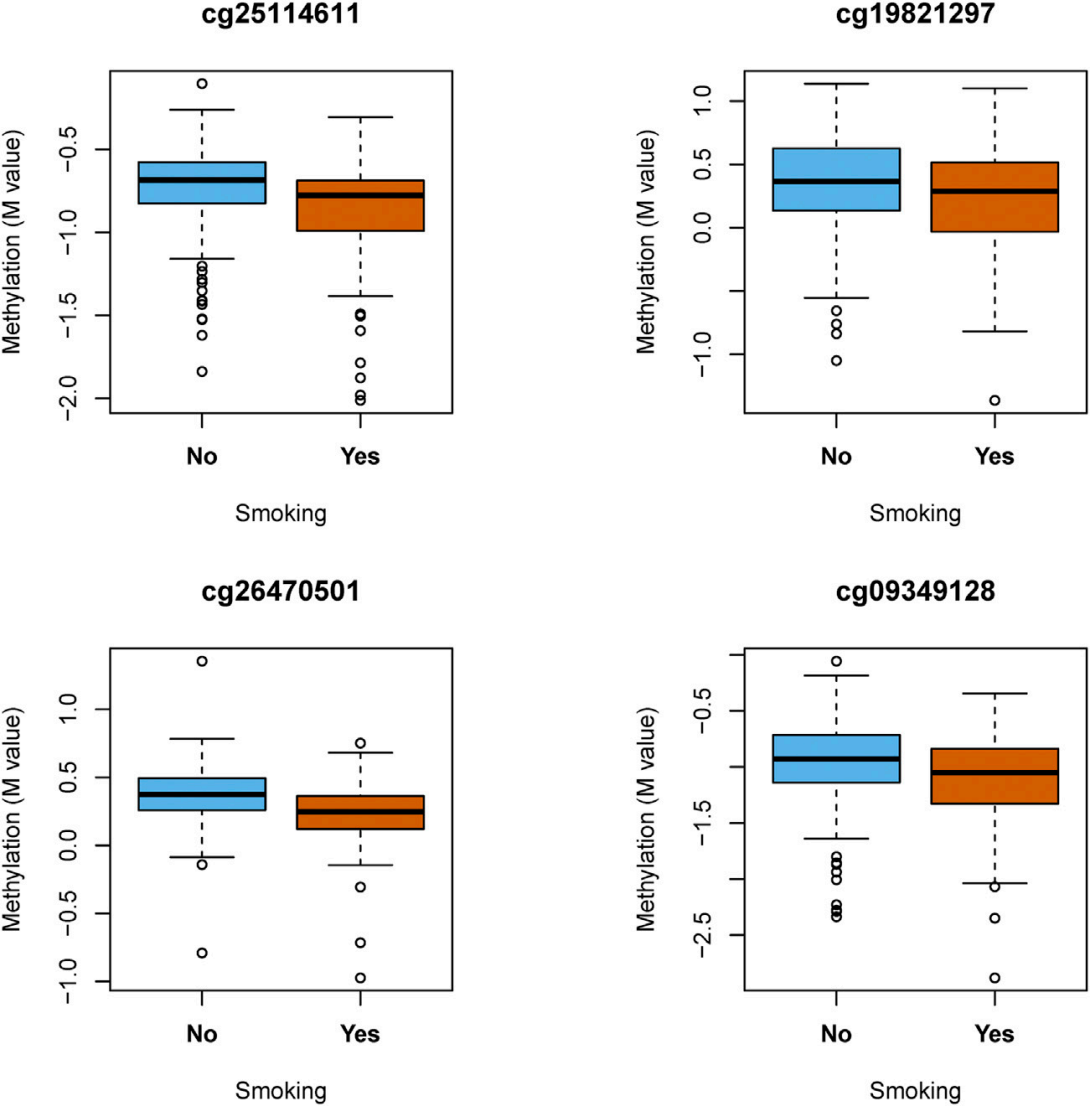
DNA methylation level of the significant CpG mediators by smoking status.

Then, we also analyzed the relationship between smoking and the above CpGs (cg04287259, cg25114611, cg10180440, cg05941027, cg19821297, cg26470501, and cg09349128), focusing on positive and negative effect sizes. As shown in Table 4, the effect of smoking was positive (cg04287259 and cg05941027), and the effect size of CpGs was negative (cg25114611, cg10180440, cg19821297, cg26470501, and cg09349128). Notably, the statistical test of the CpGs (cg25114611, cg19821297, cg26470501, and cg09349128) was significant. Notably, these four CpGs were hypomethylated in the smoking group compared to the non-smoking group (Figure 2).

**TABLE 4:** The estimation effect of smoking.

CpG	Estimation	*SE*	*t*-value	*p*
cg04287259	0.048	0.026	1.867	0.063
cg25114611	−0.106	0.031	−3.409	0.001
cg10180440	−0.05	0.032	−1.543	0.124
cg05941027	0.034	0.022	1.508	0.133
cg19821297	−0.109	0.039	−2.771	0.006
cg26470501	−0.131	0.025	−5.24	<0.001
cg09349128	−0.166	0.036	−4.599	<0.001

SE, standard error

For mediation analyses, we identified four potential CpGs (cg25114611, cg19821297, cg26470501, and cg09349128) from the above seven candidate CpGs in the mediation models, adjusting for sex, age, CD8^+^ T cells, CD4^+^ T cells, NK cells, B cells, monocytes, and granulocytes under statistical significance mediation effects (ACME, *p*-value < 0.05), which were shown in Table 5. Four CpGs showed significant ACME, with *p*-values ranging from 0.001 to 0.012 (Table 5). In Table 5, notably, the directions of ACME and ADE among these four mediator CpGs were positive. The ADE of smoking on CD was attenuated from 0.129 to 0.211 after adjusting for each mediator CpG and covariates. In a comparison with the unadjusted mediator log (OR) of 0.841 in Table 2, it is shown that adjusting for the mediator underestimated the total effect of exposure smoking on outcome CD, which was consistent with previous reports (Wang et al., 2017). The DNA methylation of cg25114611 annotated to the FKBP prolyl isomerase 5 (FKBP5) gene TSS1500, with an average mediated effect of 0.082 (95% CI: 0.030, 0.141). The cg19821297 had a mediated effect of 0.066 (95% CI: 0.014, 0.125). The cg26470501 annotated to the BCL3 transcription coactivator (BCL3) gene body, with a mediated effect of 0.118 (95% CI: 0.062, 0.181). The cg09349128 obtained a mediated effect of 0.126 (95% CI: 0.070, 0.196). The average mediation effects of smoking on CD were attenuated from 0.066 to 0.126.

**TABLE 5:** Mediation analysis on candidate CpGs between smoking and CD.

CpG	Chr	Position	Nearest gene	References gene group	ACME			ADE			Sensitivity analysis rho which ACME = 0
					Effect estimate	95% CI	*p*-value	Effect estimate	95% CI	*p*-value	
cg25114611	chr6	35,696,870	FKBP5	TSS1500	0.082	(0.030, 0.141)	0.001	0.19	(0.080, 0.295)	0.001	−0.4
cg19821297	chr19	12,890,029	—	—	0.066	(0.014, 0.125)	0.012	0.211	(0.009, 0.315)	<0.001	−0.4
cg26470501	chr19	45,252,955	BCL3	Body	0.118	(0.062, 0.181)	<0.001	0.145	(0.030, 0.255)	0.012	−0.4
cg09349128	chr22	50,327,986	—	—	0.126	(0.070, 0.196)	<0.001	0.129	(0.036, 0.238)	0.008	−0.5

FKBP5, FKBP prolyl isomerase 5; BCL3, BCL3 transcription coactivator; CI, confidence interval.

In sensitivity analyses of the mediated effect estimates, the SI assumption might be violated for residual correlations of the mediator and outcome regressions far from the observed estimated mediated effects on the above four CpGs. And then, we also conducted a sensitivity analysis on the above four CpGs to assess the robustness of our mediation analysis when the SI assumption was violated. Notably, the absolute sensitivity parameters at which ACME = 0 in the four mediator CpGs were 0.4 or 0.5, indicating that these mediation effects were robust even when the assumptions were slightly violated (Supplementary Figure S1 and Supplementary Table S1). The sensitivity analysis showed that our mediation results were relatively stable.

## Discussion

Smoking is an established risk factor for the development of CD. Our results suggest that smoking might play an important role in the well-established association of smoking and CD through DNA methylation variability. Our results also highlight the need to consider various confounding factors in epigenetic studies as a relevant biological and statistical model.

In epigenetic studies, it is crucial that DNA methylation plays a mediator role in the etiology of human diseases (Liu et al., 2013; Ventham et al., 2016; Zhang et al., 2016; Kular et al., 2018). Methylation marks are often considered potential mediators between exposures and outcomes. Numerous studies have established a clear relationship between smoking and the occurrence of IBD and its significantly detrimental effects on CD, whereas the opposite is the beneficial influence of the development of UC (Tanja Birrenbach MUBM, 2004; Khasawneh et al., 2017; Nicolaides et al., 2021; van der Sloot et al., 2021). What is less clear is whether smoking impacts the biological mechanism in CD by mediating DNA methylation.

In this article, we adopted three steps to estimate the mediation effects with high-dimensional mediator DNA methylation. We used the SIS and de-sparsified the LASSO method to reduce the dimension of potential mediators and the mediation significance test for mediation effects. Furthermore, our findings provided evidence that smoking affects CD through high-dimensional DNA methylation mediators. The results from the sensitivity test showed that the four mediator CpGs were robust when slight violation of the SI assumption was present. In our paper, we found that the differential methylation positions, such as cg26470501 (BCL3), were affected between IBD cases and controls. And the study found drastically elevated expression levels of BCL3 in CD4^+^ T cells isolated from patients with CD and UC, underlining a role for BCL3 in the pathogenesis of IBD (Reißig et al., 2017). Another study showed that the combination of glucocorticoid receptor (GR) and FKBP5 (the cg25114611 annotated to the FKBP5 gene) mutational analyses could help to identify subgroups of CD with higher chances of benefitting from glucocorticoid treatment (Maltese et al., 2012). FKBP5 revealed a significant impact on the glucocorticoid treatment response, which could result in valuable pharmacogenetic biomarkers after being confirmed in other populations and in functional studies (Skrzypczak-Zielinska et al., 2021). In addition, Tobi et al. (2018) illustrated that DNA methylation acted as a mediator of the association between prenatal adversity and risk factors for metabolic disease, and it has been shown that methylation of cg09349128 was associated with the expression of PIM3, a gene implicated in cell growth and energy metabolism (Beharry et al., 2011) and glucose-stimulated insulin secretion in β cells (Vlacich et al., 2010). In addition, the cg19821297 showed evidence of genetic influences on DNA methylation being associated with the inflammation-related epigenetic polygene (Barker et al., 2018). Furthermore, the findings that CpGs were hypomethylated provided insight into the complex interaction of genetics and epigenetics in the pathophysiology of IBD (Kalla, 2021). Besides, it has been shown that the key question was whether the hypomethylation CpG site was involved in the causal pathway (Fasanelli et al., 2015). We speculated that hypomethylation may have a crucial role in regulated inflammation.

An important strength of our causal diagram is the implementation of the counterfactual framework in mediation analysis to estimate the effects in the presence of confounders as a relevant biological model for epigenetic epidemiology. We applied screening criteria (SIS) and dimension reduction (de-sparsifying the LASSO) to select CpGs with the top largest effects and the bias-corrected regression coefficient for the outcome. Then, we conducted mediation analyses of smoking (exposure) on CD (outcome) through DNA methylation (mediator).

One limitation of the study is that our sample size for the mediation analyses is small. Using epigenome-wide significant CpG sites as candidate mediators may show stronger signals in a future study with a larger sample size. In particular, the presence of unmeasured confounders may make it impossible to distinguish causal from consequential methylation events based on observational data alone (Kang et al., 2010). Therefore, in a further study, to validate unmeasured confounding factors, we will adopt the two-step epigenetic Mendelian randomization method to estimate the mediation effect (Relton and Davey Smith, 2012).

In conclusion, this study was based on epigenetic DNA methylation data and elucidated the mechanisms related to environmental factors involved in susceptibility to IBD. Furthermore, it makes more biological sense to identify the high-dimensional mediation effect of the whole gene rather than to focus on individual methylation sites when performing a mediation analysis in an epigenetic study. Nevertheless, a statistical mediation approach might not accurately reflect the underlying causal biological mechanism. The study found that several biologically meaningful DNA methylation sites mediated the effect of smoking on CD. In future studies, the highly plausible biological mechanisms on how smoking influences CD outcome are revealed by these DNA methylation sites.

## Data Availability

The original contributions presented in the study are included in the article/Supplementary Material, further inquiries can be directed to the corresponding author.

## References

[B1] Amiri Roudbar M. Mohammadabadi M. R. Ayatollahi Mehrgardi A. Abdollahi-Arpanahi R. Momen M. Morota G. (2020). Integration of Single Nucleotide Variants and Whole-Genome DNA Methylation Profiles for Classification of Rheumatoid Arthritis Cases from Controls. Heredity 124, 658–674. 10.1038/s41437-020-0301-4 32127659 PMC7171157

[B2] Aryee M. J. Jaffe A. E. Corrada-Bravo H. Ladd-Acosta C. Feinberg A. P. Hansen K. D. (2014). Minfi: a Flexible and Comprehensive Bioconductor Package for the Analysis of Infinium DNA Methylation Microarrays. Bioinformatics 30, 1363–1369. 10.1093/bioinformatics/btu049 24478339 PMC4016708

[B3] Barker E. D. Cecil C. A. M. Walton E. Houtepen L. C. O'Connor T. G. Danese A. (2018). Inflammation-related Epigenetic Risk and Child and Adolescent Mental Health: A Prospective Study from Pregnancy to Middle Adolescence. Development Psychopathology 30, 1145–1156. 10.1017/S0954579418000330 30068408 PMC7612578

[B4] Beharry Z. Mahajan S. Zemskova M. Lin Y. W. Tholanikunnel B. G. Xia Z. (2011). The Pim Protein Kinases Regulate Energy Metabolism and Cell Growth. Proc. Natl. Acad. Sci. 108, 528–533. 10.1073/pnas.1013214108 21187426 PMC3021022

[B5] Davegårdh C. García-Calzón S. Bacos K. Ling C. (2018). DNA Methylation in the Pathogenesis of Type 2 Diabetes in Humans. Mol. Metab. 14, 12–25. 10.1016/j.molmet.2018.01.022 29496428 PMC6034041

[B6] Dezeure R. Bühlmann P. Meier L. Meinshausen N. (2014). High-dimensional Inference Confidence Intervals, P-Values and R-Software Hdi. Stat. Sci. 30, 533–558. 10.1214/15-STS527

[B7] Dick K. J. Nelson C. P. Tsaprouni L. Sandling J. K. Aïssi D. Wahl S. (2014). DNA Methylation and Body-Mass index: a Genome-wide Analysis. The Lancet 383, 1990–1998. 10.1016/S0140-6736(13)62674-4 24630777

[B8] Du P. Zhang X. Huang C. C. Jafari N. Kibbe W. A. Hou L. (2010). Comparison of Beta-Value and M-Value Methods for Quantifying Methylation Levels by Microarray Analysis. BMC Bioinformatics 11, 587. 10.1186/1471-2105-11-587 21118553 PMC3012676

[B9] Fan J. Lv J. (2008). Sure independence Screening for Ultrahigh Dimensional Feature Space. J. R. Statist. Soc. 70, 849–911. 10.1111/j.1467-9868.2008.00674.x PMC2709408 19603084

[B10] Fasanelli F. Baglietto L. Ponzi E. Guida F. Campanella G. Johansson M. (2015). Hypomethylation of Smoking-Related Genes Is Associated with Future Lung Cancer in Four Prospective Cohorts. Nat. Commun. 6, 10192. 10.1038/ncomms10192 26667048 PMC4682166

[B11] Fortin J. Labbe A. Lemire M. Zanke B. W. Hudson T. J. Fertig E. J. (2014). Functional Normalization of 450k Methylation Array Data Improves Replication in Large Cancer Studies. Genome Biol. 15, 503. 10.1186/s13059-014-0503-2 25599564 PMC4283580

[B12] Fujii R. Sato S. Tsuboi Y. Cardenas A. Suzuki K. (2021). DNA Methylation as a Mediator of Associations between the Environment and Chronic Diseases: A Scoping Review on Application of Mediation Analysis. Epigenetics 1, 1–27. 10.1080/15592294.2021.1959736 PMC9336467 34384035

[B13] Houseman E. A. Accomando W. P. Koestler D. C. Christensen B. C. Marsit C. J. Nelson H. H. (2012). DNA Methylation Arrays as Surrogate Measures of Cell Mixture Distribution. BMC Bioinformatics 13, 86. 10.1186/1471-2105-13-86 22568884 PMC3532182

[B14] Imai K. Keele L. Teppei Y. (2010). Identification, Inference and Sensitivity Analysis for Causal Mediation. Stat. Sci. 25, 51–71. 10.1214/10-STS321

[B15] Imai K. Keele L. Tingley D. (2010). A General Approach to Causal Mediation Analysis. Psychol. Methods 15, 309–334. 10.1037/a0020761 20954780

[B16] Imai K. Yamamoto T. (2013). Identification and Sensitivity Analysis for Multiple Causal Mechanisms: Revisiting Evidence from Framing Experiments. Polit. Anal. 21, 141–171. 10.1093/pan/mps040

[B17] Inoshita M. Numata S. Tajima A. Kinoshita M. Umehara H. Yamamori H. (2015). Sex Differences of Leukocytes DNA Methylation Adjusted for Estimated Cellular Proportions. Biol. Sex Differences 6. 10.1186/s13293-015-0029-7 PMC4480901 26113971

[B18] Jaffe A. E. Irizarry R. A. (2014). Accounting for Cellular Heterogeneity Is Critical in Epigenome-wide Association Studies. Genome Biol. 15, R31. 10.1186/gb-2014-15-2-r31 24495553 PMC4053810

[B19] Jenkins T. G. James E. R. Alonso D. F. Hoidal J. R. Murphy P. J. Hotaling J. M. (2017). Cigarette Smoking Significantly Alters Sperm DNA Methylation Patterns. Andrology 5, 1089–1099. 10.1111/andr.12416 28950428 PMC5679018

[B20] Joehanes R. Just A. C. Marioni R. E. Pilling L. C. Reynolds L. M. Mandaviya P. R. (2016). Epigenetic Signatures of Cigarette Smoking. Circ. Cardiovasc. Genet. 9, 436–447. 10.1161/CIRCGENETICS.116.001506 27651444 PMC5267325

[B21] Kalla R. A. A. N. J. (2021). Analysis of systemic epigenetic alterations in in ammatory bowel disease: de ning geographical, genetic, and immune-in ammatory in uences on the circulating methylome. 10.21203/rs.3.rs-537439/v1 PMC10024547 36029471

[B22] Kang E. Y. Ye C. Shpitser I. Eskin E. (2010). Detecting the Presence and Absence of Causal Relationships between Expression of Yeast Genes with Very Few Samples. J. Comput. Biol. 17, 533–546. 10.1089/cmb.2009.0176 20377462 PMC3198891

[B23] Karatzas P. S. Gazouli M. Safioleas M. Mantzarisa G. J. (2014). DNA Methylation Changes in Inflammatory Bowel Disease. Ann. Gastroenterol. 27, 125–132. 24733658 PMC3982627

[B24] Khasawneh M. Spence A. D. Addley J. Allen P. B. (2017). The Role of Smoking and Alcohol Behaviour in the Management of Inflammatory Bowel Disease. Best Pract. Res. Clin. Gastroenterol. 31, 553–559. 10.1016/j.bpg.2017.10.004 29195675

[B25] Kular L. Liu Y. Ruhrmann S. Zheleznyakova G. Marabita F. Gomez-Cabrero D. (2018). DNA Methylation as a Mediator of HLA-Drb1*15:01 and a Protective Variant in Multiple Sclerosis. Nat. Commun. 9, 2397. 10.1038/s41467-018-04732-5 29921915 PMC6008330

[B26] Lee K. W. K. Pausova Z. (2013). Cigarette Smoking and DNA Methylation. Front. Genet. 4, 132. 10.3389/fgene.2013.00132 23882278 PMC3713237

[B27] Lin Z. Hegarty J. P. Cappel J. A. Yu W. Chen X. Faber P. (2011). Identification of Disease-Associated DNA Methylation in Intestinal Tissues from Patients with Inflammatory Bowel Disease. Clin. Genet. 80, 59–67. 10.1111/j.1399-0004.2010.01546.x 20950376

[B28] Liu Y. Aryee M. J. Padyukov L. Fallin M. D. Hesselberg E. Runarsson A. (2013). Epigenome-wide Association Data Implicate DNA Methylation as an Intermediary of Genetic Risk in Rheumatoid Arthritis. Nat. Biotechnol. 31, 142–147. 10.1038/nbt.2487 23334450 PMC3598632

[B29] Maltese P. Palma L. Sfara C. de Rocco P. Latiano A. Palmieri O. (2012). Glucocorticoid Resistance in Crohn's Disease and Ulcerative Colitis: an Association Study Investigating GR and FKBP5 Gene Polymorphisms. Pharmacogenomics J. 12, 432–438. 10.1038/tpj.2011.26 21788965

[B30] McDermott E. Ryan E. J. Tosetto M. Gibson D. Burrage J. Keegan D. (2015). DNA Methylation Profiling in Inflammatory Bowel Disease Provides New Insights into Disease Pathogenesis. J. Crohn's Colitis 10, 77–86. 10.1093/ecco-jcc/jjv176 26419460 PMC5013897

[B31] Nicolaides S. Vasudevan A. Long T. van Langenberg D. (2021). The Impact of Tobacco Smoking on Treatment Choice and Efficacy in Inflammatory Bowel Disease. Intestinal Res. 19, 158–170. 10.5217/ir.2020.00008 PMC8100381 33040518

[B32] Reißig S. Tang Y. Nikolaev A. Gerlach K. Wolf C. Davari K. (2017). Elevated Levels of Bcl-3 Inhibits Treg Development and Function Resulting in Spontaneous Colitis. Nat. Commun. 8, 15069. 10.1038/ncomms15069 28452361 PMC5414353

[B33] Relton C. L. Davey Smith G. (2012). Two-step Epigenetic Mendelian Randomization: a Strategy for Establishing the Causal Role of Epigenetic Processes in Pathways to Disease. Int. J. Epidemiol. 41, 161–176. 10.1093/ije/dyr233 22422451 PMC3304531

[B34] Severs M. van Erp S. J. H. van der Valk M. E. Mangen M. J. J. Fidder H. H. van der Have M. (2016). Smoking Is Associated with Extra-intestinal Manifestations in Inflammatory Bowel Disease. J. Crohn's Colitis 10, 455–461. 10.1093/ecco-jcc/jjv238 26721937 PMC4946753

[B35] Sharp G. C. Arathimos R. Reese S. E. Page C. M. Felix J. Küpers L. K. (2018). Maternal Alcohol Consumption and Offspring DNA Methylation: Findings from Six General Population-Based Birth Cohorts. Epigenomics 10, 27–42. 10.2217/epi-2017-0095 29172695 PMC5753623

[B36] Shu C. Justice A. C. Zhang X. Wang Z. Hancock D. B. Johnson E. O. (2020). DNA Methylation Mediates the Effect of Cocaine Use on HIV Severity. Clin. Epigenetics 12. 10.1186/s13148-020-00934-1 PMC7491141 32928285

[B37] Skrzypczak-Zielinska M. Gabryel M. Marszalek D. Dobrowolska A. Slomski R. (2021). NGS Study of Glucocorticoid Response Genes in Inflammatory Bowel Disease Patients. Arch. Med. Sci. 17, 417–433. 10.5114/aoms.2019.84470 33747278 PMC7959014

[B38] Tanja Birrenbach Mubm (2004). Inflammatory Bowel Disease and Smoking a Review of Epidemiology, Pathophysiology, and Therapeutic Implications. Inflamm. Bowel Dis. 10, 848–859. 10.1097/00054725-200411000-00019 15626903

[B39] Tingley D. Yamamoto T. Hirose K. Keele L. Imai K. (2014). Mediation: R Package for Causal Mediation Analysis.

[B40] Tobi E. W. Slieker R. C. Luijk R. Dekkers K. F. Stein A. D. Xu K. M. (2018). DNA Methylation as a Mediator of the Association between Prenatal Adversity and Risk Factors for Metabolic Disease in Adulthood. Sci. Adv. 4, o4364. 10.1126/sciadv.aao4364 PMC5792223 29399631

[B41] Tsaprouni L. G. Yang T. Bell J. Dick K. J. Kanoni S. Nisbet J. (2014). Cigarette Smoking Reduces DNA Methylation Levels at Multiple Genomic Loci but the Effect Is Partially Reversible upon Cessation. Epigenetics 9, 1382–1396. 10.4161/15592294.2014.969637 25424692 PMC4623553

[B42] van de Geer S. Bühlmann P. Ritov Y. A. Dezeure R. (2014). On Asymptotically Optimal Confidence Regions and Tests for High-Dimensional Models. Ann. Stat. 42, 1166–1202. 10.1214/14-AOS1221

[B43] van der Sloot K. W. J. Tiems J. L. Visschedijk M. C. Festen E. A. M. van Dullemen H. M. Weersma R. K. (2021). Cigarette Smoke Increases Risk for Colorectal Neoplasia in Inflammatory Bowel Disease. Clin. Gastroenterol. Hepatol. S1542-3565 (21), 00018–5. 10.1016/j.cgh.2021.01.015 33453400

[B44] VanderWeele T. Vansteelandt S. (2014). Mediation Analysis with Multiple Mediators. Epidemiologic Methods 2, 95–115. 10.1515/em-2012-0010 25580377 PMC4287269

[B45] Ventham N. T. Kennedy N. A. Adams A. T. Kalla R. Heath S. O'Leary K. R. (2016). Integrative Epigenome-wide Analysis Demonstrates that DNA Methylation May Mediate Genetic Risk in Inflammatory Bowel Disease. Nat. Commun. 7, 13507. 10.1038/ncomms13507 27886173 PMC5133631

[B46] Vlacich G. Nawijn M. C. Webb G. C. Steiner D. F. (2010). Pim3 Negatively Regulates Glucose-Stimulated Insulin Secretion. Islets 2, 308–317. 10.4161/isl.2.5.13058 21099329 PMC3025049

[B47] Wahl S. Drong A. Lehne B. Loh M. Scott W. R. Kunze S. (2017). Epigenome-wide Association Study of Body Mass index, and the Adverse Outcomes of Adiposity. Nature 541, 81–86. 10.1038/nature20784 28002404 PMC5570525

[B48] Wang T. Li H. Su P. Yu Y. Sun X. Liu Y. (2017). Sensitivity Analysis for Mistakenly Adjusting for Mediators in Estimating Total Effect in Observational Studies. BMJ Open 7, e15640. 10.1136/bmjopen-2016-015640 PMC5719285 29162569

[B49] Wu D. Yang H. Winham S. J. Natanzon Y. Koestler D. C. Luo T. (2018). Mediation Analysis of Alcohol Consumption, DNA Methylation, and Epithelial Ovarian Cancer. J. Hum. Genet. 63, 339–348. 10.1038/s10038-017-0385-8 29321518 PMC5985822

[B50] Zhang H. Zheng Y. Zhang Z. Gao T. Joyce B. Yoon G. (2016). Estimating and Testing High-Dimensional Mediation Effects in Epigenetic Studies. Bioinformatics 32, 3150–3154. 10.1093/bioinformatics/btw351 27357171 PMC5048064

[B51] Zhang X. Hu Y. Aouizerat B. E. Peng G. Marconi V. C. Corley M. J. (2018). Machine Learning Selected Smoking-Associated DNA Methylation Signatures that Predict HIV Prognosis and Mortality. Clin. Epigenetics 10. 10.1186/s13148-018-0591-z PMC6293604 30545403

