# Supplementary figures and images for: High-Dimensional DNA Methylation Mediates the Effect of Smoking on Crohn’s Disease
Source: Front Genet. 2022 Apr 5;13:831885. doi: 10.3389/fgene.2022.831885 (PMC9016182; doi:10.3389/fgene.2022.831885)

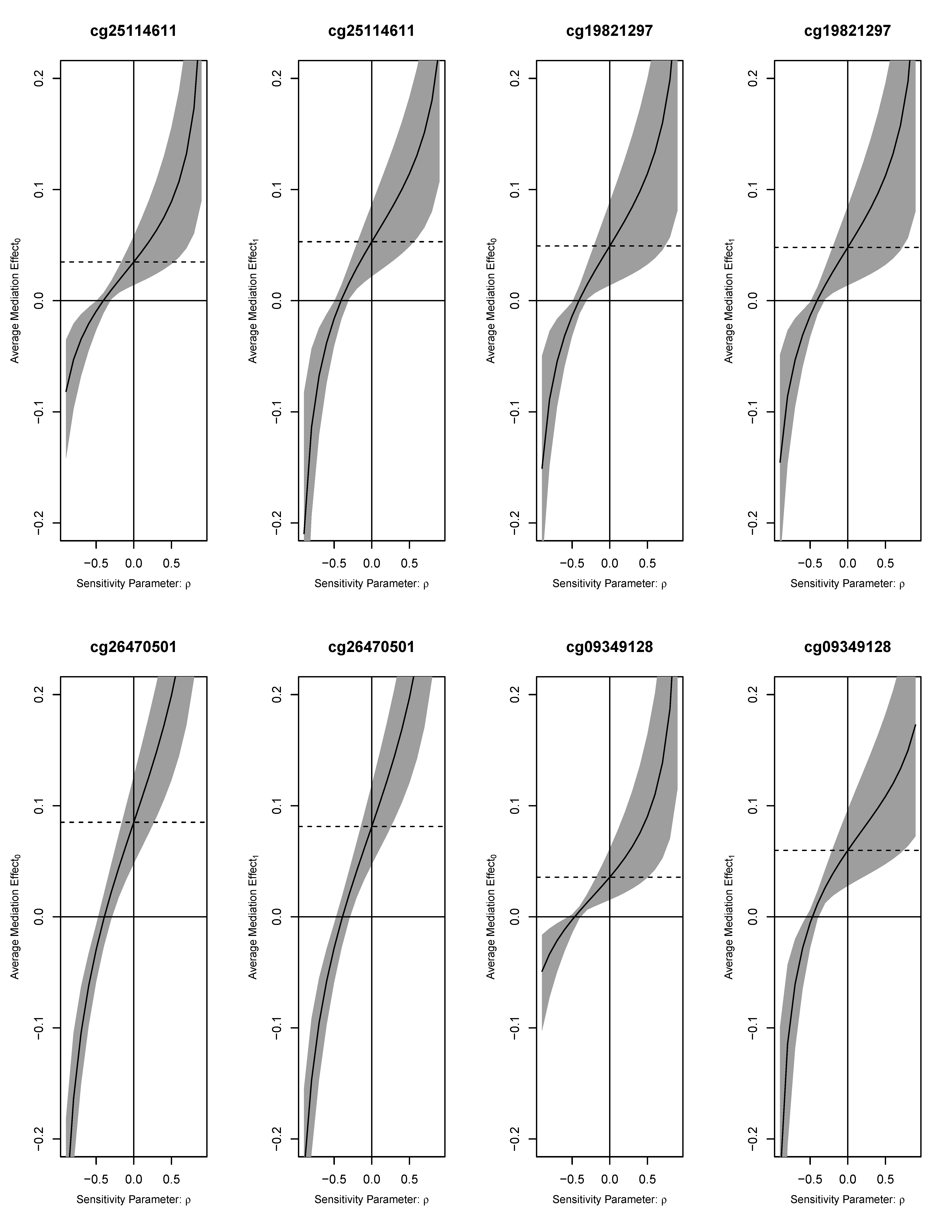

Supplement: Supplementary file 1 [file Image1.tif]
