# Supplementary material for: High-Dimensional DNA Methylation Mediates the Effect of Smoking on Crohn’s Disease
Source: Front Genet. 2022 Apr 5;13:831885. doi: 10.3389/fgene.2022.831885 (PMC9016182; doi:10.3389/fgene.2022.831885)
Supplement: Supplementary file 2 [file Table1.doc]

Supplementary Table 1: The sensitivity parameter ρ of the 95% confidence interval for ACME coverage zero

| CpG | Chr | Position | Nearest gene | Reference gene group | Sensitivity analysis  rho which  ACME = 0 | *CI* lower | *CI* upper |
| --- | --- | --- | --- | --- | --- | --- | --- |
| cg25114611 | chr6 | 35696870 | FKBP5 | TSS1500 | -0.4 | -0.011 | 0.007 |
| cg19821297 | chr19 | 12890029 | - | - | -0.4 | -0.011 | 0.014 |
| cg26470501 | chr19 | 45252955 | BCL3 | Body | -0.4 | -0.027 | 0.020 |
| cg09349128 | chr22 | 50327986 | - | - | -0.5 | -0.015 | 0.002 |
